# The potential risks of delaying the second vaccine dose during the SARS-CoV-2 pandemic

**DOI:** 10.1093/qjmed/hcab046

**Published:** 2021-03-02

**Authors:** I R Humphreys, A J Godkin

**Affiliations:** From the Division of Infection and Immunity, School of Medicine, Cardiff University, Henry Wellcome Building, Heath Park, Cardiff CF144XN, UK

Prevention of infectious diseases through vaccination is one of the greatest success stories of medicine, with a 200 year old history. Although vaccinology is a branch of the science of immunology, most successful vaccines have been developed empirically. In contrast, the rapid development of vaccines to offer protection from SARS-CoV-2 infection- a huge global collective effort- has combined rational design with new highly responsive platform technologies. Leading vaccine candidates have focused mainly on inducing neutralizing antibody responses to SARS-CoV-2 spike protein, include nucleic acid (RNA) vaccines (e.g. Pfizer, Moderna) and replication-deficient Adenovirus vectors (e.g. Oxford-AstraZeneca), expressing the modified spike gene (although whole inactivated SARS-CoV-2/subunit vaccines have also been used elsewhere). These vaccines tested in phase II/III studies offer protection from infection for at least 3–6 months, probably longer. Based on the data from these carefully designed randomized control trials, mass population vaccination programs are commencing, with highest risk subjects, such as the elderly or individuals with co-morbidities, being first prioritized.

For priming CD4^+^ (and CD8^+^) T-cells, and stimulation of B cells, transient exposure to antigen is sufficient to induce an antigen-dependent program of proliferation and differentiation.^1^ The strength and duration of antigen exposure, plus additional signals through co-stimulation, influences the differentiation process and determines the functional qualities of the effector and memory lymphocytes that develop. Although the exact details of antigen kinetics and co-stimulation may be hazy in many vaccination studies, the empirical result of repeated vaccination is to increase the number of protective antigen-specific T and B lymphocytes over a critical frequency, reflected usually by significantly higher titer of relevant antibodies.

The protocols for both RNA and Adenoviral SARS-CoV-2 vaccines use similar approaches, a prime followed by a second injection boost, 21–28 days apart. The dose and schedule of vaccination profoundly influences functional anti-SARS-CoV-2 antibody production, generally exhibiting at least a 10-fold increase in neutralization antibody titers along with enhanced vaccine-induced T cell immunity.

Thus, scientists and clinicians have rapidly developed and tested vaccines based more on rational design than pure empiricism, and the results are highly encouraging. All the vaccines induce protective immune responses with, to date, slightly better efficacy in RNA-based vaccines. The vast bulk of data and knowledge regarding protective efficiency is generated on prime/boost schedules as outlined above (e.g. 21–28 days apart). It is, therefore, understandable that there is some consternation amongst doctors and the general public that the timing of the second booster injection has been extended upto 12 weeks in the UK, driven by logistical reasons (i.e. not enough vaccines to go around currently) but not based on much scientific evidence; after these measures were introduced, some data have recently emerged in pre-print format suggesting the second boost can be delayed using the Oxford vaccine.^2^ It is likely that a 12-week boost will work well from what we know of immune kinetics, but is there a risk in leaving this 3 month gap? There are three types of risk to counterbalance: (i) not vaccinating high-risk subjects in a timely fashion, (ii) if one dose is ineffectual or suboptimal, it may leave the individual at risk until the booster given and (iii) partial protection may actually facilitate the SARS-CoV-2 virus to mutate *in situ*, escaping the weak immune responses and encourage vaccine-resistant virus variants, which could then spread.

**Table 1.** Summary of immunological and efficacy data of one vs. two doses of the Pfizer, Moderna and Oxford-AstraZeneca SARS-CoV-2 vaccines

Vaccine	Vaccine dosing	T cell induction	Antibody titers	Neutralizing antibody	Efficacy (symptomatic infection) (%)	Efficacy (severe disease) (%)
Pfizer (mRNA)	Single	+	++	+	50	90
	Double	+++	+++	+++	95	90
Moderna (mRNA)	Single	+	++	+	94 (after d14)	100
	Double	+++	+++	+++	94	100
AstraZeneca (adenovirus)	Single	+++ (d14)	++	++	>64–77	100 (>d22)
	Double	++	+++	+++	70.4–90	100

Notes: Efficacy data refers to >14 days after the final vaccine dose. Immunogenicity data is expressed in relative terms within the individual assay in each study, no comparison between different studies can be made: + responses not much greater than baseline; ++ responses significantly above baseline; +++ responses substantially above baseline.

Consider some details of the trials carried out so far. Phase III efficacy trials of the Pfizer RNA-based vaccine examined immunogenicity and efficacy of a prime-boost regime with a 21-day space between the two injections. Boosting the response led to a log-fold increase in IgG titers and dramatically increased antibody efficacy measured in an *in vitro* neutralization assay.^3^ Similarly, analysis of T cell responses post-boost revealed induction of spike receptor binding domain (RBD)-specific T_H_1 and CD8^+^ T-cell responses in >95% and >75% of individuals, respectively. Weaker T cell induction was observed after a single dose vaccination.^3^ The Moderna mRNA-1273 vaccine behaves in the same manner, inducing much higher neutralizing antibody titers after the second vaccination boost at day 28.^4^ The Oxford/AstraZeneca adenovirus-based vaccine again exhibits increased antibody responses after boosting, albeit with a negligible impact on T cells.^5^

Given this reported reduction in immunogenicity of a single-dose vaccine, what level of protection does this induce? In initial trials, a single dose of Pfizer vaccine afforded ∼50% and ∼90% protection from infection and severe disease, respectively.^6^ Real-World data derived from Israel (>500 000 individuals) also indicated ∼50% reduction in SARS-CoV-2 infection from 13 days post-single dose.^7^ Interestingly, however, early reported efficacy (∼94%) of the Moderna mRNA-based vaccine was afforded as early as 14 days after the first dose.^8^ The Oxford/AstraZeneca vaccine offered >64% efficacy after one dose as compared to 70.4–90% efficacy of two doses (depending on dosing regime). Thus, once these SARS-CoV-2 vaccines have developed initial immunity, a substantial level of protection is afforded, albeit reduced to varying degrees.^9^ Whether this protection wanes beyond the tested boosting time-points examined in these clinical trials, however, is a large (and important) unknown.

Thus, the level of protection, at least in the short term, is reasonable, albeit not perfect, after the prime dose of these vaccines. However, a far more serious and insidious consequence may be the acceleration of vaccine redundancy. Immune pressure can promote virus evolution, and SARS-CoV-2-specific antibody drives the selection of viruses capable of avoiding spike RBD-specific immunity *in vitro*.^10^ Notably, substitutions within the RBD of the spike glycoprotein reported in SARS-CoV-2 strains in the human population (e.g. K417N/T, E484K and N501Y) reduce protection conferred by plasma derived from convalescents or vaccines,^11^ demonstrating that the development of vaccine-resistant SARS-CoV-2 has commenced. The rapid emergence of a SARS-CoV-2 variant that is resistant to current vaccines would spell a new disaster. As events unfurl daily, the recent news that the Oxford vaccine is being halted in South Africa due to loss of efficacy demonstrates the rapidly changing landscape.

In summary, it is not a trivial decision to alter the evidence-based vaccination schedule, and although we may get away with it, there is a risk that it may increase the chance of virulent mutations of SARS-CoV-2 to emerge. In this transitional phase of the pandemic, scientific advisors and politicians have to be vigilant of these issues. Widespread immune monitoring of individuals pre-/post-vaccination will provide the sort of objective data that at the moment we simply lack. In the meantime, establishing and maintaining control of virus transmission during this vaccine rollout is important to reduce the risks of developing vaccine-resistant SARS-CoV-2 strains.

*Conflict of interest*. None declared.
